# PDK4 inhibits osteoarthritis progression by activating the PPAR pathway

**DOI:** 10.1186/s13018-024-04583-5

**Published:** 2024-02-02

**Authors:** Zhengnan Li, Lifeng Xie, Hui Zeng, Yaohong Wu

**Affiliations:** 1 Department of Sports Medicine, Ganzhou People’s Hospital, No.16, MeiGuan Road, Zhanggong District, Ganzhou City, 341000 Jiangxi Province China; 2 Department of Orthopedics, The Second Affiliated Hospital of Nanchang University, No.1 MinDe Road, Donghu District, Nanchang City, 330000 Jiangxi Province China; 3 Department of Spine Surgery, Ganzhou People’s Hospital, No.16, MeiGuan Road, Zhanggong District, Ganzhou City, 341000 Jiangxi Province China

**Keywords:** Osteoarthritis, PDK4, Inflammatory cytokines, ECM degradation, PPAR pathway

## Abstract

**Background:**

Osteoarthritis (OA) is a degenerative joint disease caused by the deterioration of cartilage. However, the underlying mechanisms of OA pathogenesis remain elusive.

**Methods:**

Hub genes were screened by bioinformatics analysis based on the GSE114007 and GSE169077 datasets. The Sprague–Dawley (SD) rat model of OA was constructed by intra-articular injection of a mixture of papain and L-cysteine. Hematoxylin–eosin (HE) staining was used to detect pathological changes in OA rat models. Inflammatory cytokine levels in serum were measured employing the enzyme-linked immunosorbent assay (ELISA). The reverse transcription quantitative PCR (RT-qPCR) was implemented to assess the hub gene expressions in OA rat models. The roles of PDK4 and the mechanism regulating the PPAR pathway were evaluated through western blot, cell counting kit-8 (CCK-8), ELISA, and flow cytometry assays in C28/I2 chondrocytes induced by IL-1β.

**Results:**

Six hub genes were identified, of which COL1A1, POSTN, FAP, and CDH11 expressions were elevated, while PDK4 and ANGPTL4 were reduced in OA. Overexpression of PDK4 inhibited apoptosis, inflammatory cytokine levels (TNF-α, IL-8, and IL-6), and extracellular matrix (ECM) degradation protein expressions (MMP-3, MMP-13, and ADAMTS-4) in IL-1β-induced chondrocytes. Further investigation revealed that PDK4 promoted the expression of PPAR signaling pathway-related proteins: PPARA, PPARD, and ACSL1. Additionally, GW9662, an inhibitor of the PPAR pathway, significantly counteracted the inhibitory effect of PDK4 overexpression on IL-1β-induced chondrocytes.

**Conclusion:**

PDK4 inhibits OA development by activating the PPAR pathway, which provides new insights into the OA management.

**Supplementary Information:**

The online version contains supplementary material available at 10.1186/s13018-024-04583-5.

## Introduction

Osteoarthritis (OA) is a progressive degenerative disease, presenting symptoms like pain and impaired joint function [1]. OA impacts one in three individuals over the age of 65, and its increasing prevalence poses a substantial economic burden [2]. Factors contributing to the induction of OA encompass age, joint injury, obesity, and genetic predisposition [3]. The progression of OA is regulated by chondrocytes and the inflammatory response [4]. Currently, the mainstay of treatment for OA involves in the medications, such as analgesics and nonsteroidal anti-inflammatory drugs, and surgical procedures, which can alleviate pain and improve joint function, but do not have a clinically significant effect on curing OA [5]. Hence, delving deeper into the precise pathogenesis of OA is crucial to enhance the outcome of OA treatments.

Pyruvate dehydrogenase kinase 4 (PDK4), a member of the pyruvate PDKs family, is essential in the management of cellular energy metabolism [6]. It is extensively expressed in diverse tissues, such as skeletal muscle, heart, pancreatic islets, liver, and kidneys [7]. PDK4 is implicated in the development of multiple illnesses, including tumors [8], subarachnoid hemorrhage [9], myocardial injuries [10], and muscle atrophy [11]. Furthermore, a growing number of researches have revealed that there may be a significant correlation between PDK4 and OA. In rheumatoid arthritis, exosomal microRNA-106b derived from synovial fibroblasts suppresses chondrocyte proliferation and migration by regulating PDK4[12]. PDK4 is linked to lipid metabolism in the skeletal muscle [13]. Moreover, insulin signaling in skeletal muscle during obesity can be inhibited by PDK4 through promotion of endoplasmic reticulum–mitochondrial contact [14]. SIK3 mediates chondrocyte maturation in endochondral bone formation by decreasing PDK4 expression levels and increasing acetyl-CoA levels [15]. Nevertheless, the functions and underlying molecular mechanisms of PDK4 in OA still remain unclear.

The peroxisome proliferator-activated receptor (PPAR) is a member of the nuclear receptor superfamily and is a ligand-activated transcription factor consisting of three PPARα, PPARγ, and PPARβ/δ distinct subtypes [16]. By modulating several signaling pathways, PPARs regulate articular cartilage homeostasis and attenuate inflammatory responses in OA [17]. Moreover, recent investigations have shown that by inhibiting pyroptosis, PPAR-γ activation inhibits OA development via the Nrf2/NLRP3 and PGC-1 α/Δψ m Pathways [18]. Deficiency of PPARγ leads to severe and accelerated OA, which is associated with abnormal mTOR signaling in the articular cartilage [19]. All these studies demonstrated that the PPAR pathway has an important role in OA. Importantly, a study has demonstrated that PPAR β/δ-interfering peptide promotes immunomodulatory properties of mesenchymal stromal cells via reducing the expression level of the PPARβ/δ target gene PDK4 [20]. Therefore, it is important to explore the correlation between PDK4 and the PPAR pathway in OA.

In this investigation, based on the GSE114007 and GSE169077 datasets, PDK4 was identified as a key gene for OA by bioinformatics analysis and its low expression was verified by reverse transcription quantitative PCR (RT-qPCR) in the rat OA model. Subsequently, the function of PDK4 in OA and its regulation of the PPAR pathway was explored in the IL-1β-induced chondrocyte model. The aim of this investigation was to provide new insights and possible treatment approaches for the OA.

## Materials and methods

### Screening and processing of datasets

OA-related datasets were selected by entering the keyword "osteoarthritis" in the Gene Expression Omnibus (GEO) database (http://www.ncbi.nlm.nih.gov/geo). Subsequently, the GSE114007 and GSE169077 datasets were enrolled in this study. The dataset GSE114007 comprises 18 control cartilage samples and 20 OA cartilage samples. Additionally, GSE169077 dataset includes 5 control cartilage samples and 6 OA cartilage samples. GEO2R tool (www.ncbi.nlm.nih.gov/geo/geo2r) was employed to identify DEGs between control and OA samples in the two datasets according to the *P* < 0.05 and |logFC|≥ 2. Volcano maps and heatmaps of the DEGs were then generated utilizing the ggplot2 and pheatmap packages in R software (version 4.2.3). The common DEGs in the GSE114007 and GSE169077 datasets were determined using the Venn diagram tool (https://bioinfogp.cnb.csic.es/tools/venny/index.html).

### Functional enrichment analysis of DEGs

The DAVID (https://david.ncifcrf.gov/; version 6.8) was utilized to conduct Gene Ontology (GO) and Kyoto Encyclopedia of Genes and Genomes (KEGG) analysis with the threshold set at *P* < 0.05. The top 5 cellular component (CC), biological process (BP), and molecular function (MF) categories were presented as a bubble plot, and the top 8 enriched pathways of KEGG were displayed as a histogram via the ggplot2 package in the R software.

### Protein–protein interaction (PPI) network construction and hub genes identification

In the STRING (https://string-db.org/) database, the PPI network of the DEGs was constructed with an interaction score > 0.4. Then, the PPI network was imported into the Cytoscape software (version 3.7.0) for visualization. Important modules in the PPI network were identified using the Molecular Complex Detection (MCODE) plug-in in the Cytoscape software. The default parameters were as follows: degree cutoff = 2, node score cutoff = 0.2, k-score = 2, and max. depth = 100. The Degree algorithm in the cytoHubba plug-in of Cytoscape software was applied to score the genes of each node, and the top 12 genes with the highest scores were filtered out. Combining MCODE and degree algorithms, the hub genes were finally identified.

### Hub genes analysis

Principal component analysis (PCA), correlation, and expression distribution, GO, analyses were conducted based on the GSE114007 dataset. PCA was executed with the factoextra package in R software, utilizing hub gene expression levels as variables. Hub gene correlations were assessed with the corrplot and Hmisc packages. For visualizing key gene expression distribution, ridgeline maps were created with the ggridges package. GO analysis for hub genes was conducted by the circlize package. Additionally, the receiver operating characteristic (ROC) curve was plotted by the pROC package in the GSE114007 and GSE169077 datasets to evaluate the diagnostic value of hub genes for OA.

### Construction of OA rat model

All experimental protocols were executed according to guidelines in the Animal Experimental Welfare Committee of the Institute of Animal Science. Experimental procedures involving animal care and use in this study were approved by the Ganzhou People’s Hospital (TY-DKY2023-002-01). A total of 12 male Sprague–Dawley (SD) rats (6 weeks old, 200–220 g) were obtained from SPF Biotechnology Co., Ltd. (Beijing, China). All rats were housed in conditions free of pathogens, with a 12-h light/dark cycle (22 ± 2 °C), and they were free access to food and water for a week before to the experiment.

All SD rats were randomly allocated to a control group (6 per group) and an OA group (6 per group). A 5% papain solution was prepared by mixing it with 0.03 mol/L L-cysteine in a 1:1 ratio. For the OA group, the right thigh of rats was immobilized to allow the right leg to be vertically bent upward and protrude into the joint cavity. Subsequently, 0.2 mL prepared mixed solution was injected vertically into the joint cavity of the rats on the 1th, 4th, and 7th days. The control group was administered an equal amount of saline injected into the right knee joint only. After 6 weeks of model construction, rats were deeply anesthetized with 2% isoflurane and through an inhalation anesthesia machine for 2–3 min, and then, articular cartilage tissue and rat serum were extracted from each group of rats for subsequent experiments.

### Hematoxylin–eosin (HE) staining and safranin O/fast green staining

The rat cartilage tissues were fixed in 4% paraformaldehyde solution. Subsequently, the samples were decalcified in a 10% ethylenediaminetetraacetic acid (EDTA) solution for 3 weeks, followed by dehydration through a graded alcohol series. The samples underwent embedding in paraffin and were cut into 5 μM slices. Subsequently, tissue slices were contaminated with hematoxylin–eosin (HE) stain and safranin O/fast green stain. Following this, the slices were analyzed using a microscope (Olympus, Tokyo, Japan). For HE staining, the chondrocyte nuclei appeared blue, and other tissues exhibited a pink color. For safranin O/fast green staining, normal cartilage showed red, and the background displayed green. The degree of cartilage degeneration was assessed according to the Osteoarthritis Research Society International (OARSI) scoring system.

### Enzyme-linked immunosorbent assay (ELISA)

The levels of Tumor necrosis factor-alpha (TNF-α), interleukin-8 (IL-8), and interleukin-6 (IL-6) in the rat serum were quantified using an ELISA kit (Esebio Biotechnology Co., Ltd., Shanghai, China) according to the manufacturer’s instructions. The microplate reader (DALB, Shanghai, China) was utilized to measure the optical density (OD) at a wavelength of 450 nm.

### RNA extraction and reverse transcription quantitative PCR (RT-qPCR)

Extraction of total RNA from rat tissues were performed using TRIzol reagent (Thermo Fisher Scientific, Massachusetts, USA) and reverse-transcribed to cDNA with The RevertAid First Strand cDNA Synthesis Kit (Thermo Fisher Scientific). RT-qPCR was performed with the SYBR Premix Ex Taq (Takara, Dalian, China) on a 7500 real-time PCR System (Thermo Fisher Scientific) following the manufacturer’s protocol. Subsequently, the relative mRNA levels were quantified with the 2^−ΔΔCt^ method. The primers employed in this study are provided in Additional file 1: Table S1.

### Cell culture and processing

Human C28/I2 chondrocytes were purchased from Icellbioscience Biotechnology Co., Ltd. (Shanghai, China). C28/I2 chondrocytes were cultured in DMEM/F12 with 10% fetal bovine serum (Thermo Fisher Scientific) and 1% penicillin–streptomycin at 37 °C with 5% CO_2_. Chondrocytes were induced using 10 ng/mL IL-1β (Sigma-Aldrich, Missouri, USA) for 24 h to induce OA cell model. The PDK4 coding sequence was inserted into the pcDNA3.1 vector plasmid to establish the chondrocyte model of PDK4 overexpression. Subsequently, the plasmid was transfected into the chondrocytes with Lipofectamine® 3000 reagent (Thermo Fisher Scientific) as per the guidelines provided by the manufacturer. The empty vector plasmid (ov-NC) was utilized as a control. Moreover, in the rescue experiments, cells were treated with the PPAR signaling pathway inhibitor GW9662 (10 μM) for 24 h to explore the correlation between PDK4 and PPAR pathway in OA.

### Cell counting kit-8 (CCK-8) assay

Proliferative capacity of chondrocytes in each group was assessed by the CCK-8 method at five time points: 0 h, 24 h, 48 h, 72 h, and 96 h post-transfection. A total of 1 × 10^5^ cells from each group were seeded into 96-well plates. Chondrocytes were cultured at 37 °C until 80% confluence, and then, 10 μL of CCK-8 solution (Solarbio, Beijing, China) was introduced into each well. After a 2-h incubation, absorbance at 450 nm was determined by a microplate reader (DALB).

### Detection of apoptosis by flow cytometry

Chondrocyte apoptosis was quantified by an annexin V-fluorescein isothiocyanate (FITC)/propidium iodide (PI) cell apoptosis kit (Thermo Fisher Scientific). Chondrocytes were treated with Annexin V-FITC (50 μg/mL) and PI (10 μg/mL) at 37 °C for 10 min. Following treatment, the labeled cells were assessed for apoptosis with a FACScan flow cytometer (Becton, Dickinson and Company, New Jersey, USA).

### Western blot assay

Radioimmunoprecipitation assay (RIPA) solution encompassing protease inhibitors (Solarbio) was used to lysate total cell proteins. The proteins were then transferred to polyvinylidene difluoride (PVDF) membranes (Roche, Basel, Switzerland) after being separated on an SDS–polyacrylamide gel. At room temperature, the membranes were blocked with 5% fat-free dry milk before being cultivated with primary antibodies with diluted 2000 times. The membranes were then treated with the secondary antibody for 2 h at room temperature. ECL-enhanced chemiluminescence reagents (Amersham, Little Chalfont, UK) were used to visualize protein bands. Primary antibodies used in this investigation included anti-PDK4 (ab110336; Abcam, Cambridge, UK), anti-MMP-3 (ab52915; Abcam), anti-MMP-13 (ab39012; Abcam), anti-ADAMTS-4 (ab314856; Abcam), anti-PPARA (ab227074; Abcam), anti-PPARD (ab178866; Abcam), anti-ACSL1 (ab177958; Abcam), and anti-GAPDH (ab52915; Abcam).

### Statistical analysis

All statistical analyses were performed using GraphPad Prism 8 software, and continuous variables are presented as the mean ± SD from three independent experiments. An unpaired Student’s t-test was used to measure differences between two groups. For comparisons between multiple means, one-way ANOVA was used, followed by Tukey’s test. A statistically significant difference was defined as *P* < 0.05. **Results**

### DEGs identification in OA

**Fig. 1:**
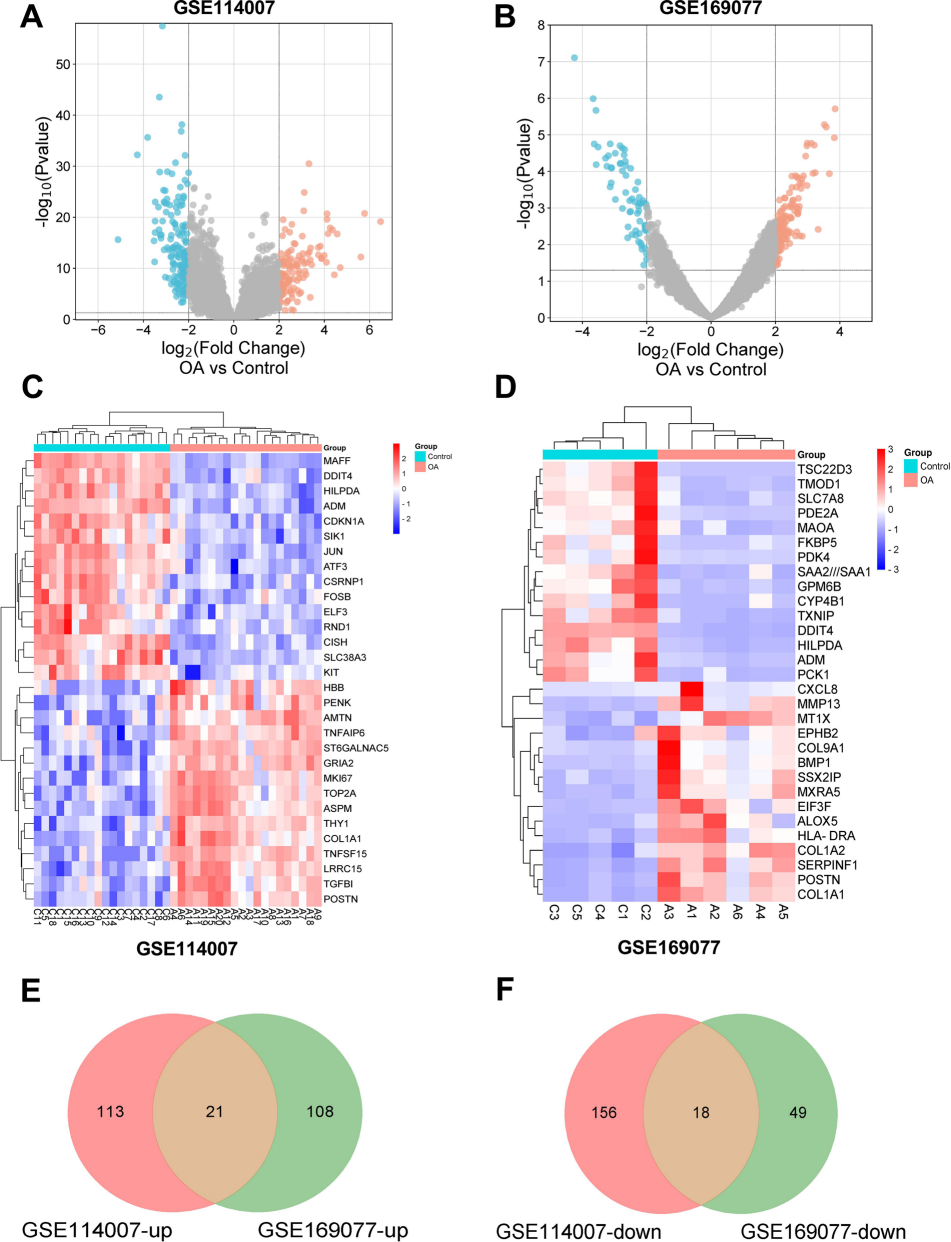
Screening DEGs in GSE114007 and GSE169077 datasets. **A**, **B** Volcano plots illustrating DEGs in the GSE114007 and GSE169077 datasets. The horizontal axis represents log2FoldChange, and the vertical axis represents -log10 (p-value). Red dots indicate up-regulated genes, while blue dots indicate down-regulated genes in the respective groups. **C** and **D** Heatmaps display the top 15 up-regulated and down-regulated genes in the GSE114007 and GSE169077 datasets. Genes are presented horizontally, with one column per sample. Red indicates highly expressed genes, while blue indicates lowly expressed genes. **E**, **F** Venn diagrams for common up-regulated and down-regulated DEGs in both datasets. The numerical values in each circle denote the number of differential genes identified in that specific dataset. The overlapping portion of the circles represents the common DEGs shared between the two datasets

Standardization and data correction were performed on the selected samples in the GSE114007 and GSE169077 datasets, respectively. The findings revealed that the samples were appropriately centered, indicating that both datasets were suitable for subsequent analysis (Additional file 2: Figures S1A and S1B). In the GSE114007 dataset, 308 DEGs were screened, comprising 134 highly expressed and 174 lowly expressed genes (Fig. 1A). The GSE169077 dataset contained 196 DEGs, containing 129 up-regulated and 67 down-regulated genes (Fig. 1B). The top 15 DEGs for both up- and down-regulation were then selected in each dataset (Additional file 1: Table S2 and Additional file 1: Table S3), and heatmaps were generated for visualization (Fig. 1C and D). Subsequently, the Venn diagram was employed to identify common DEGs between the two datasets, revealing 21 common up-regulated genes and 18 common down-regulated genes (Fig. 1E and F).

### GO and KEGG analysis of DEGs

GO and KEGG functional analyses were performed for the 39 common DEGs. The GO enrichment analysis results were categorized into MF, BP, and CC. Significant GO term entries were selected for plotting the enrichment analysis bubble (Additional file 2: Figure S2A). Notably, the DEGs exhibited significant enrichment in categories such as cell adhesion, extracellular matrix (ECM) organization, ECM structural constituent, and proteinase binding. Significant KEGG pathways were determined according to the p-value, and the enrichment analysis histogram was generated for visualization (Additional file 2: Figure S2B). The DEGs were prominently engaged in pathways such as protein digestion and absorption, PI3K-Akt pathway, PPAR pathway, and diabetic cardiomyopathy.

**Fig. 2:**
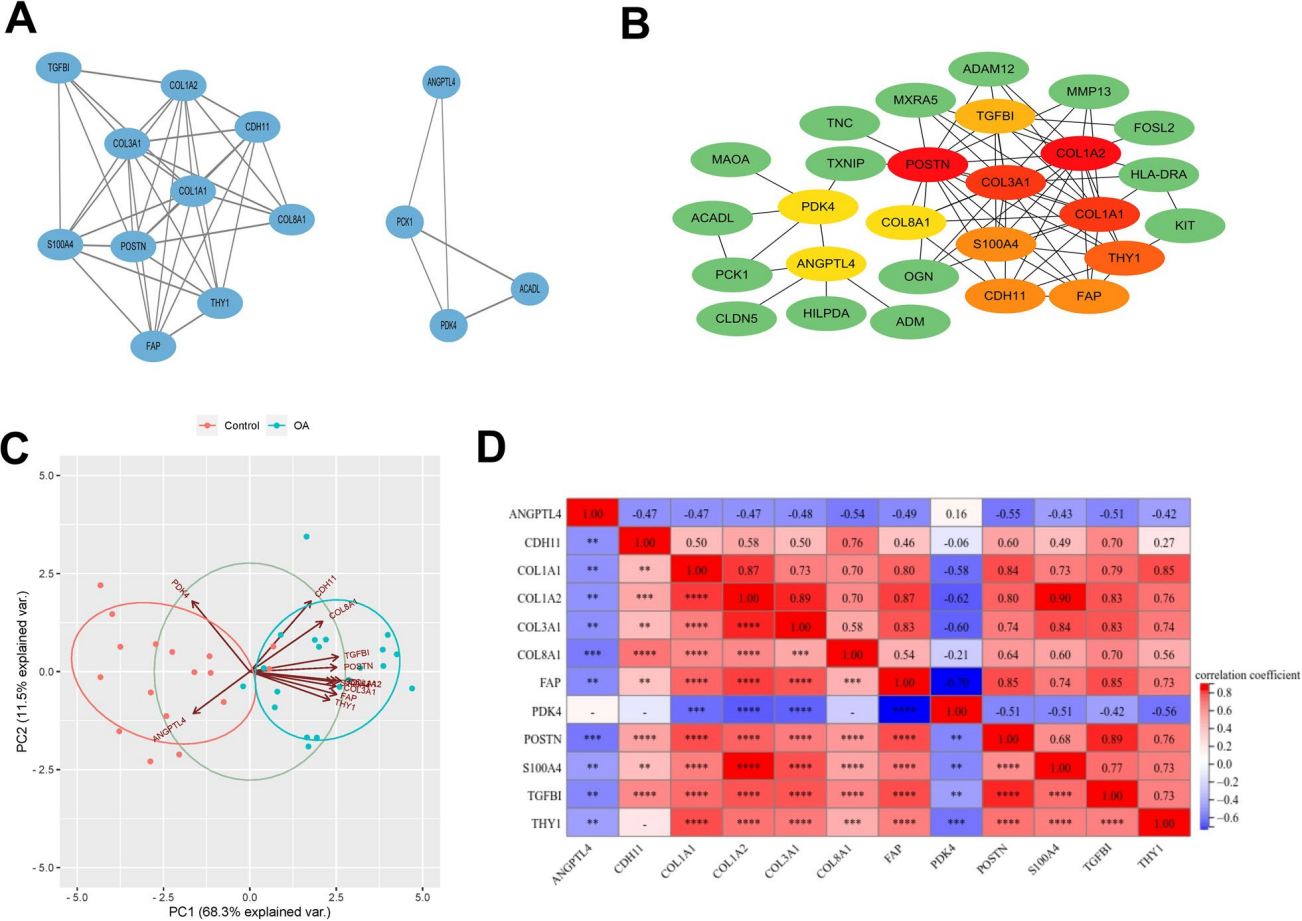
Through the MCODE algorithm and degree algorithm in CytoHubba plug-in to identify key genes in the PPI network. **A** Two significant modules identified based on MCODE analysis. The lines between the nodes represent the interactions between the genes. **B** The top 12 genes with the highest scores were filtered based on the degree algorithm in cytoHubba plug-in. The lines between the nodes represent the interactions between the genes. **C** PCA of the key genes. The axes PC1 and PC2 represent the first and second principal components, indicating the explanatory rate of potential variables for differences. Dots represent samples, and different colors signify distinct groupings. **D** Matrix correlation analysis of key genes

### Hub gene screening and identification

A PPI network was established based on 39 DEGs using the STRING tool (Additional file 2: Figure S3). The PPI network model was visualized through Cytoscape software. Subsequently, the MCODE algorithm was employed for analysis, revealing the two most important modules (Fig. 2A). The degree algorithm in CytoHubba plug-in was employed to screen the top 12 genes (Fig. 2B). Combining the both methods, a total of 12 hub genes were identified: COL1A2, POSTN, COL3A1, COL1A1, THY1, FAP, S100A4, CDH11, TGFBI, COL8A1, PDK4, and ANGPTL4.

### Hub gene analysis

The PCA resulted in two axes: PC1, explaining 68.3% of the variance, and PC2, which accounted for 11.5% of the variance (Fig. 2C). We then plotted heat maps of correlations between key genes and ridgeline maps of gene expression distributions (Fig. 2D and Additional file 2: Figure S4A). In addition, GO enrichment analysis showed that key genes were mainly correlated with blood vessel development and ECM regulation (Additional file 2: Figure S4B). ROC curve analysis showed that the AUC values of these 12 key genes were all greater than 0.8 in the GSE114007 and GSE169077 datasets, indicating that they could distinguish normal individuals from OA patients (Additional file 2: Figure S5A-5L and Additional file 2: Figure S6A-L).

### Construction of OA rat model and validation of hub gene expression

**Fig. 3:**
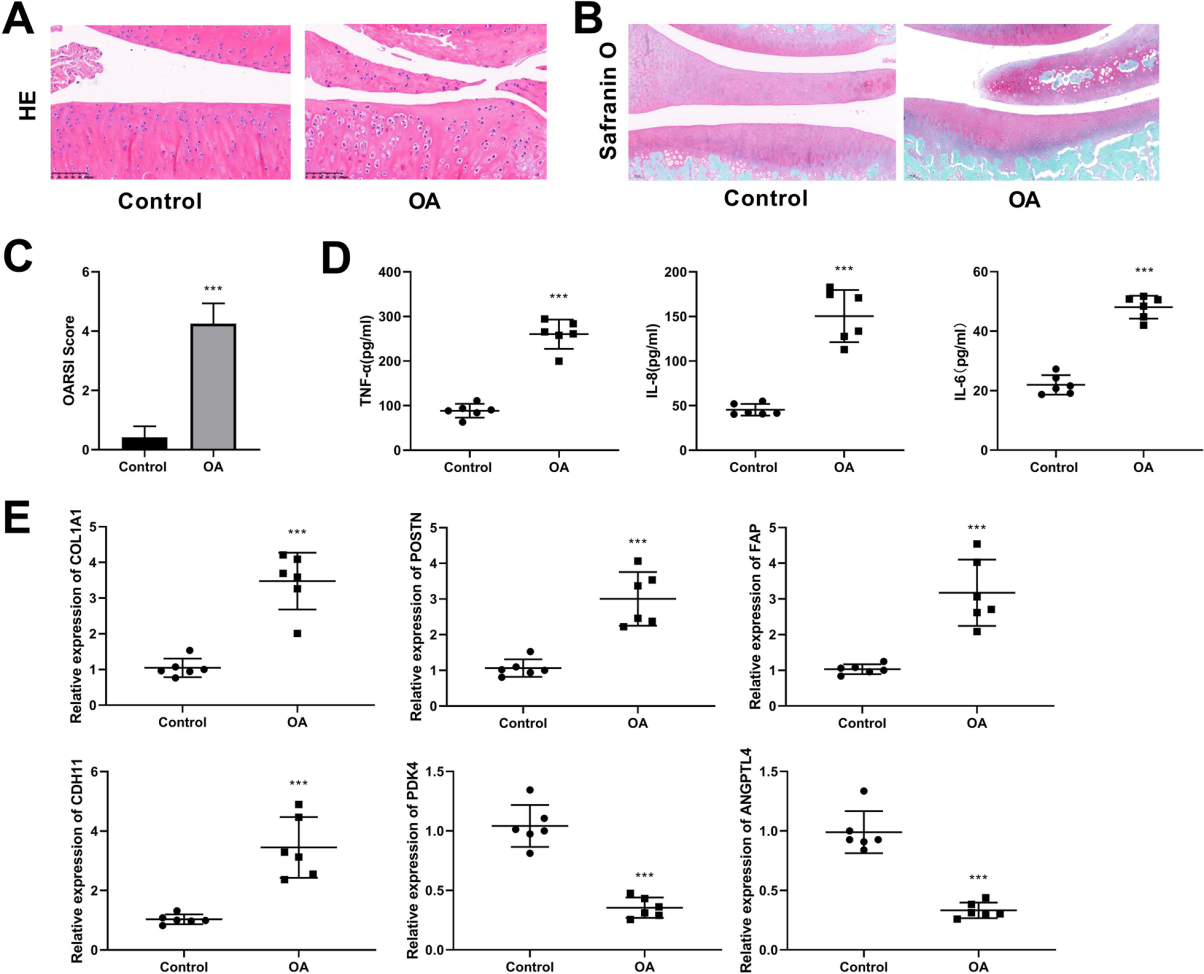
Establishment of the OA rat model and confirmation of hub gene expression. **A**, **B** Representative images of HE staining and Safranin O/fast green staining for articular cartilage (magnification: × 100; scale bar: 100 μM). **C** OARSI scores of cartilage specimens from control and OA groups. **D** Serum levels of inflammatory factors (TNF-α, IL-8, and IL-6) were detected by ELISA. **E** RT-qPCR was used to measure hub gene expressions in OA rats. ***P < 0.001 versus control group

To further explored the expression of hub genes, the OA rat model was established. The morphology and structure of the cartilage tissue in OA rats were analyzed through HE staining. The HE staining results revealed that in the control group, chondrocytes displayed a neat arrangement, the matrix was uniformly distributed, and no inflammatory cell infiltration was found. In contrast, the OA group exhibited a considerable decrease in normal chondrocytes, disorganized chondrocytes, and a notable increase in inflammatory cell (Fig. 3A). Safranin O/fast green staining showed that the OA model group exhibited fewer positive staining in the cartilage layer and severe cartilage degeneration compared to the control group (Fig. 3B). The OARSI scores of the OA group were significantly higher than those of the control group (Fig. 3C). Furthermore, ELISA assay demonstrated that the concentrations of TNF-α, IL-8, and IL-6 were substantially elevated in the OA group in comparison with the control group (Fig. 3D). Then the expression of the top 4 increased and top 2 decreased key genes with higher scores was detected by RT-qPCR. The findings proved that the mRNA expressions of COL1A1, POSTN, FAP, and CDH11 were notably up-regulated, while the PDK4 and ANGPTL4 were considerably down-regulated in the OA model group in contrast to the control group (Fig. 3E). Given the relatively limited exploration of PDK4 in the OA, it was selected for in-depth investigation.

### PDK4 inhibits apoptosis, inflammatory response, and ECM degradation in IL-1β-induced C28/I2 chondrocytes

**Fig. 4:**
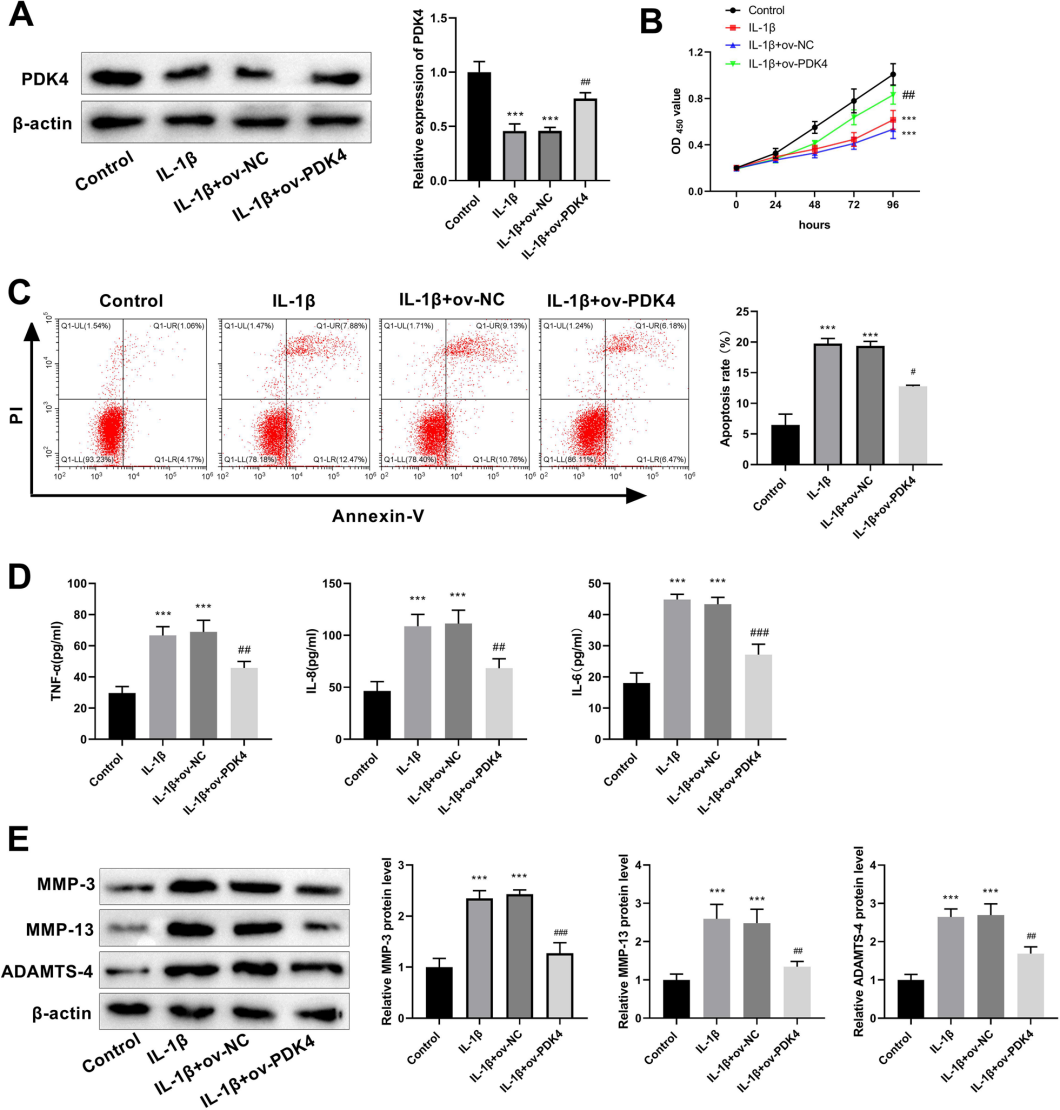
PDK4 suppresses apoptosis, inflammatory response, and ECM degradation in IL-1β-induced C28/I2 chondrocytes. **A** Western blot was used to detect the overexpression efficiency of PDK4. **B** OD values (450 nm) were measured by CCK-8 to assess the viability of chondrocytes in each group. **C** The rate of apoptosis was assessed using flow cytometry. **D** ELISA was employed to measure the levels of TNF-α, IL-8, and IL-6. **E** The expression of proteins associated with ECM degradation, including MMP-3, MMP-13, and ADAMTS-4, was assessed using western blot analysis. ***P < 0.001 versus control group; ^##^*P* < 0.01, ^###^*P* < 0.001 versus IL-1β + ov-NC group

Subsequently, we established the in vitro cell model OA by inducing C28/I2 chondrocytes with IL-1β to explore the regulatory functions of PDK4 in the OA. Western blot showed that the PDK4 protein expression was down-regulated in the OA chondrocyte model group. Moreover, in contrast to the IL-1β + ov-NC group, the protein expression of PDK4 was increased in the IL-1β + ov-PDK4 group (Fig. 4A). Results from CCK8 suggested that IL-1β reduced C28/I2 chondrocytes viability, which was restored after PDK4 overexpression (Fig. 4B). Flow cytometry indicated an increased chondrocyte apoptosis with IL-1β treatment, and PDK4 overexpression alleviated this effect (Fig. 4C). In addition, levels of inflammatory factors, including TNF-α, IL-8, and IL-6 were measured using ELISA. IL-1β induction led to a significant increase in these factors, and PDK4 overexpression suppressed their levels (Fig. 4D). The expression of ECM degradation-related proteins (MMP-3, MMP-13, and ADAMTS-4) were detected by western blot. The results showed that protein expression of MMP-3, MMP-13, and ADAMTS-4 were increased in the IL-1β treatment group compared with the control group. Conversely, these proteins were decreased in IL-1β + ov-PDK4 group relative to IL-1β + ov-NC group (Fig. 4E).

### PDK4 promotes the PPAR pathway in IL-1β-induced C28/I2 chondrocytes

**Fig. 5:**
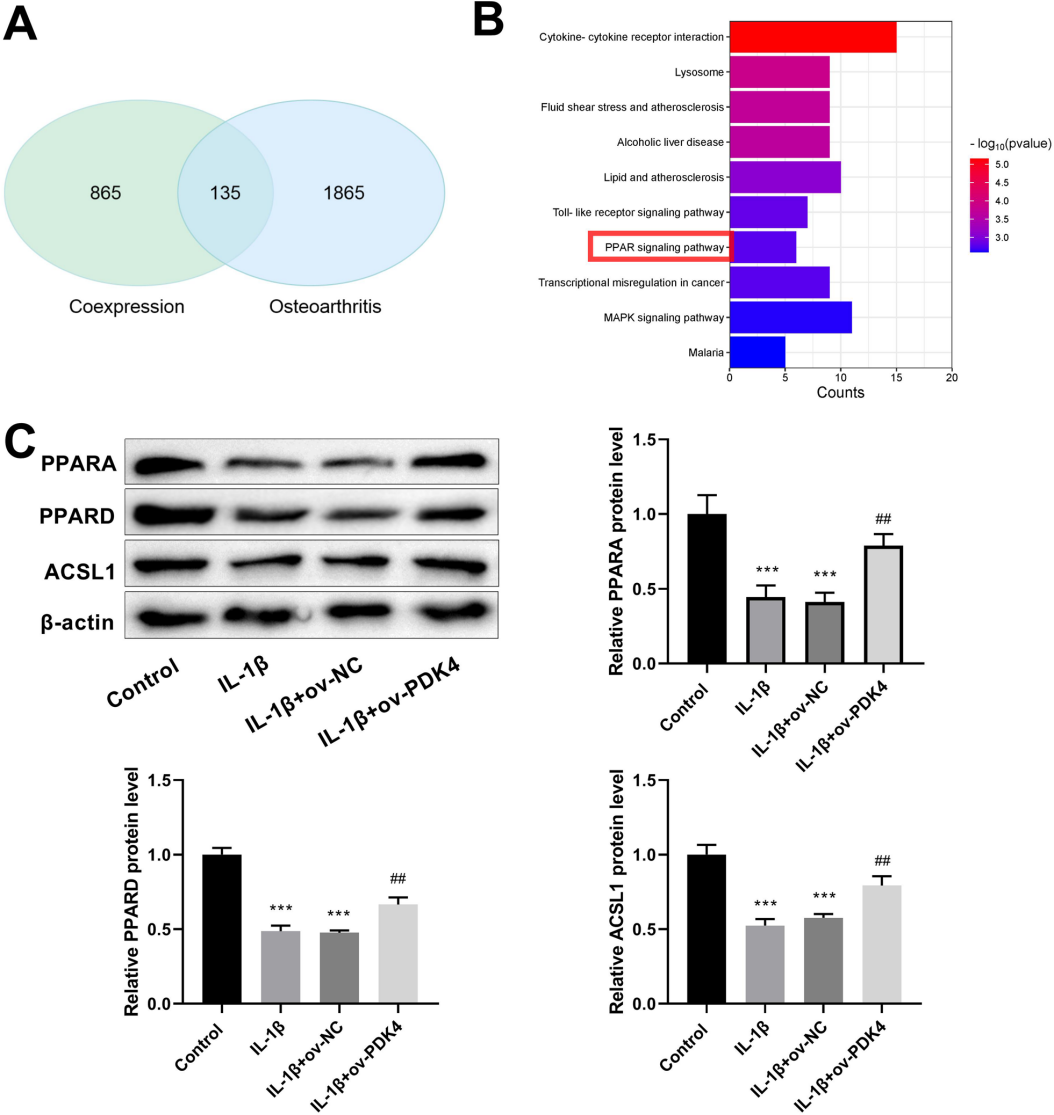
PDK4 regulates the PPAR signaling pathway in IL-1β-induced chondrocytes. **A** Venn diagram of PDK4 co-expressed genes (top 1000) and OA-associated genes (top 2000). **B** KEGG enrichment analysis of common genes. **C** Western blot analysis was implemented to examine the expression of proteins related to the PPAR signaling pathway, including PPARA, PPARD, and ACSL1. ***P < 0.001 versus control group; ^##^*P* < 0.01 versus IL-1β + ov-NC group

Subsequently, we explored the downstream signaling pathways regulated by PDK4. The top 1000 PDK4 co-expressed genes were extracted from the Coxpresdb database (https://coxpresdb.jp/), and OA-related genes (top 2000) were obtained from GeneCards database (https://www.genecards.org/). Venn diagram analysis identified 135 common genes (Fig. 5A), and KEGG enrichment analysis indicated that these genes were chiefly significantly correlated with the PPAR pathway (Fig. 5B). Western blot analysis was carried out to measure PPAR pathway-related proteins: PPARA, PPARD, and ACSL1 (Fig. 5C) to investigate the influence of PDK4 on the PPAR pathway. The findings confirmed that IL-1β processing suppressed the PPAR pathway-related protein expressions, whereas PDK4 overexpression enhanced these protein expressions in the PPAR pathway.

### PDK4 hinders the progression of OA by promoting the PPAR signaling pathway

**Fig. 6:**
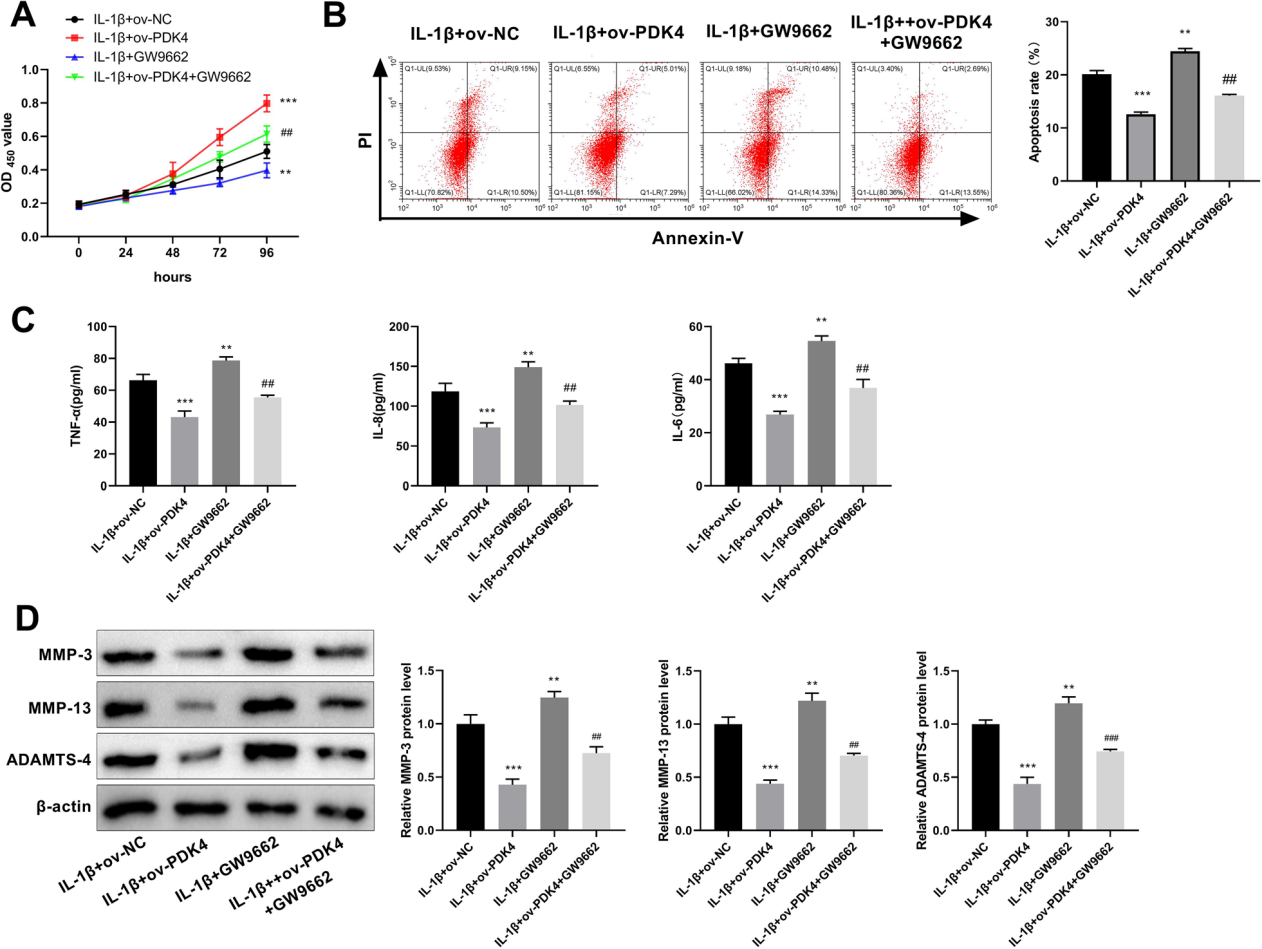
PDK4 inhibits IL-1β-induced chondrocyte apoptosis, inflammation, and ECM degradation by promoting the PPAR pathway. **A** IL-1β-induced chondrocyte viability was assessed by CCK-8 after PDK4 overexpression and GW9662 treatment. **B** Flow cytometry was performed to evaluate apoptosis in IL-1β-treated chondrocytes in four groups. **C** TNF-α, IL-8, and IL-6 levels were measured using ELISA in IL-1β-induced chondrocytes. **D** Western blot analysis was used to measure the expression of proteins linked to ECM degradation, including as MMP-3, MMP-13, and ADAMTS-4 after PDK4 overexpression and GW9662 treatment in IL-1β-induced chondrocytes. **P < 0.01, ***P < 0.001 versus IL-1β + ov-NC group; ^##^*P* < 0.01, ^###^*P* < 0.001 versus IL-1β + ov-PDK4 group

To investigate whether PDK4 affected OA progression through the PPAR signaling pathway, feedback experiments were performed using a PPAR pathway inhibitor (GW9662). The CCK8 assay revealed a higher cell viability in the IL-β + ov-PDK4 group compared to the IL-β + ov-NC group, while the IL-β + GW9662 group exhibited lower cell viability. Meanwhile, cell viability was reduced in the IL-β + ov-PDK4 + GW9662 group compared to the IL-β + ov-PDK4 group, suggesting that the inhibitor of PPAR signaling pathway reversed the role of PDK4 overexpression in OA to some extent (Fig. 6A). Flow cytometry indicated that overexpression of PDK4 inhibited cell apoptosis, whereas treatment with GW9662 promoted apoptosis comparison with control group. Remarkably, the addition of GW9662 in the PDK4 overexpression treatment group significantly reversed the inhibitory effect of PDK4 on apoptosis (Fig. 6B). ELISA results revealed that GW9662 further amplified the increase in inflammatory factor levels (TNF-α, IL-8, and IL-6) induced by IL-1β administration. Simultaneously, it substantially reversed the inhibitory effect of PDK4 overexpression on inflammatory factors (Fig. 6C). Additionally, GW9662 reduced the inhibitory effect of PDK4 overexpression on ECM degradation-associated protein expression (MMP-3, MMP-13, and ADAMTS-4) protein expression (Fig. 6D).

## Discussion

OA is a prevalent contributor to global disability, which is typically associated with osteophyte formation, articular cartilage degeneration, ligament and knee joint deterioration, and synovial inflammation [21]. OA is a complicated disease affected by many factors and its precise pathogenesis remains unclear. In this investigation, based on a comprehensive bioinformatics analysis of the GSE114007 and GSE169077 datasets, six key genes were identified and had the potential capacity to distinguish between control individuals and those with OA. Further functional exploration uncovered that PDK4 played a role in inhibiting apoptosis, inflammation levels, and ECM degradation in IL-1β-induced C28/I2 chondrocytes, and these effects were mediated through the activation of the PPAR pathway.

Inflammatory cytokine compounds are important in the OA pathogenesis. Chondrocyte apoptosis, joint inflammation, and cartilage ECM degradation are all regulated by IL-1β [22, 23]. TNF-α is highly expressed in synovial fluid, synovium, cartilage, and subchondral bone layers [24]. It induces similar inflammatory responses with IL-1β in various cells and can be as a susceptibility marker for OA [25]. IL-6 is a major cytokine involved in alterations in the subchondral bone layer that is detectable in synovial fluid and expressed in osteoarthritic cartilage, making its suppression an appealing prospective target for OA treatment [26]. Furthermore, IL-6 and IL-8 production is induced by IL-1β and TNF-α, and these cytokines can further enhance inflammation and cartilage destruction initiated by IL-1 or TNF-α [27]. In the present investigation, we constructed rat model of OA with papain and L-cysteine and found that the expressions of TNF-α, IL-6, and IL-8 were elevated in the OA model, which is consistent with previous investigations [28, 29].

Subsequently, the six hub gene expression levels were detected by RT-qPCR in the OA rat model. The findings indicated that the expression levels of COL1A1, POSTN, FAP, and CDH11 were elevated, whereas the PDK4 and ANGPTL4 were lowered. COL1A1 (Collagen 1A1) participates in the synthesis of the essential proteins in connective tissues, including skin, bone, ligaments, and tendons [30]. Mutations in the COL1A1 and COL1A2 genes can lead to osteogenesis imperfecta inherited in an autosomal dominant manner [31]. Moreover, COL1A1 is highly expressed in OA, and recent study has revealed that fruit peels protect cartilage in the rat model of sodium iodoacetate-induced OA by down-regulating COL1A1(20, 21). POSTN is a matricellular protein that contains glutamate [32]. Increased levels of POSTN in both cartilage and bone are linked to OA. Its loss-of-function inhibits the development of age-related spontaneous OA [33]. FAP (fibroblast activation protein) is a serine protease, contributing to the degradation of denatured type I collagen, α2-antiprotease, and FGF21. In OA, there is an up-regulation of FAP expression, and the inhibition of its expression mitigates cartilage matrix degradation and slows down OA progression [34, 35]. CDH11, also known as Cadherin-11, is a type II classical cadherin that was first identified in the osteoblasts of mice [36]. CDH11 alleviates OA by regulating Wnt/β-catenin pathway, which is mediated by exosomes released from bone marrow mesenchymal stem cells [37]. ANGPTL4 belongs to the angiopoietin-like family [38]. It is involved in the progression of osteolytic disease, intervertebral disc degeneration, and rheumatoid arthritis [39–41]. In OA, ANGPTL4 silencing reduces inflammation, ECM degradation, and apoptosis in OA through the Sirtuin 1/NF-κB pathway [42]. These investigations have demonstrated the important role of COL1A1, POSTN, FAP, CDH11, and ANGPTL4 in the development of OA, and COL1A1, POSTN, FAP, and CDH11 may act as promoters of OA, while ANGPTL4 appears to act as a protective factor. Among the six key genes, PDK4 captured our attention, particularly because it is rarely reported in the OA. Consequently, we chose PDK4 for in-depth exploration.

Inflammatory cytokines affect the production of chondrocyte synthetic MMP and ADAMTS enzymes, which are destructive to cartilage components [43]. TNF-α, IL-6, IL-8, MMP-3, MMP-13, and ADAMTS-4 expression was elevated in vitro model of OA induced by IL-1β, which was inhibited by PDK4 overexpression in this study. MMP-3 promotes vascularization to cartilage and inflammatory cell aggregation [44]. MMP-13 is the major MMP implicated in cartilage degradation via cleaving type II collagen, which is a potential target for the OA therapy [45]. ADAMTS-4 is engaged in cartilage degradation and the cleavage of the Glu373-Ala374 bond within the IGD domain of aggrecan [46]. In addition, both inflammatory cytokines and MMP induce apoptosis in chondrocytes [47, 48]. In the present investigation, we found that IL-1β treatment notably promoted chondrocyte apoptosis, while overexpression of PDK4 countered this effect. Furthermore, PDK4 regulates glucose and fatty acid metabolism, allowing cells to adapt to changes in nutrient supply and energy demand [49]. Previous investigation has shown that TNF-α and IL-1β influence the efficiency of the respiratory chain and reduce ATP production in chondrocyte mitochondria [50]. Therefore, we hypothesize that there is an important correlation between PDK4, energy metabolism, inflammatory response, ECM degradation, and apoptosis, which further regulates the progression of OA.

Subsequently, we investigated the underlying molecular mechanisms by which PDK4 affected OA development and found that there might be an important connection between PDK4 and PPAR signaling pathway. The western blot results indicated that PDK4 promoted PPAR signaling pathway-related protein expressions, including PPARA, PPARD, and ACSL1. Moreover, GW9662, an inhibitor of the PPAR signaling pathway, largely inverted the inhibitory impact of PDK4 overexpression on apoptosis, inflammatory response, and ECM degradation in chondrocytes. These results suggest that the PPAR pathway is a downstream pathway regulated by PDK4 in OA. On the other hand, GW9662 effectively reduces key proteins in the PPAR pathway rather than completely inhibiting them [51], indicating that GW9662 treatment significantly reverses the role of PDK4 in OA without completely blocking it. PPARs are critical for controlling inflammation and energy homeostasis, and it has been demonstrated that PPARs are involved in the progression of OA homeostasis [52]. In human OA cartilage explants, the PPARα agonist Wy-14643 inhibits inflammatory and destructive reactions, potentially through decreasing MMP-1, MMP-3, and MMP-13 mRNA expression in IL-1β-responsive cartilage explants [53]. The inhibition of IL-1β-induced inflammation, apoptosis, and ECM degradation in chondrocytes by knocking down FABP4 is mediated through the activation of PPARγ [54]. In our investigation, we found that PDK4 inhibited apoptosis, inflammation, and ECM degradation through activation of the PPAR signaling pathway in IL-1β-induced chondrocytes. Furthermore, free fatty acids are endogenous PPARα agonists and can indirectly lead to elevated PDK expression in fasting or diabetic conditions [55]. The high-fat diet increases the expression of PPAR-γ, subsequently promoting the expression of PDK4 and regulating fatty acid oxidation and ketogenesis [56]. These findings suggest that PDK4, a metabolism-related gene, may serve as a link between the PPAR signaling pathway, inflammatory response, ECM degradation, and apoptosis in OA.

## Conclusion

In the present investigation, we identify six key genes in OA, in which the expression of COL1A1, POSTN, FAP, and CDH11 are increased, whereas the expression of PDK4 and ANGPTL4 are decreased. More importantly, PDK4 inhibits apoptosis, inflammation levels, and ECM degradation by activating the PPAR pathway in IL-1β-induced chondrocytes. This study offers a novel perspective on the pathogenesis and potential therapeutic strategies of OA.

### Supplementary Information

**Additional file 1.** Primer sequences and top 15 up- and down-regulated DEGs in the GSE114007 and GSE169077 datasets.

**Additional file 2.** Supplementary figures for bioinformatics analysis of DEGs.

## Data Availability

The datasets generated and/or analyzed during the current study are available from the corresponding author on reasonable request.
