# Supplementary material for: PDK4 inhibits osteoarthritis progression by activating the PPAR pathway
Source: J Orthop Surg Res. 2024 Feb 2;19:109. doi: 10.1186/s13018-024-04583-5 (PMC10835968; doi:10.1186/s13018-024-04583-5)
Supplement: Supplementary file 1 — Additional file 1. Primer sequences and top 15 up- and down-regulated DEGs in the GSE114007 and GSE169077 datasets. [file 13018_2024_4583_MOESM1_ESM.docx]

**Supplementary table 1 Primer sequences used in this study**

| Primer Name | Sequence (5'-3') |
| --- | --- |
| GAPDH-F | CTCATGACCACAGTCCATGC |
| GAPDH-R | TTCAGCTCTGGGATGACCTT |
| COL1A1-F | TGAACGTGACCAAAAACCAA |
| COL1A1-R | GCAGAAAAGGCAGCATTAGG |
| POSTN -F | AACCAAGGACCTGAAACACG |
| POSTN -R | GTGTCAGGACACGGTCAATG |
| FAP -F | TTCGGTGTTTTGCATCTGAA |
| FAP -R | AGCCACAGACTCTGCCAGTT |
| CDH11-F | CACAGGATGGTGTGGTGAAG |
| CDH11-R | AGGCTCATCGGCATCTTCTA |
| PDK4-F | GCCTTGGGAGAAATGTGTGT |
| PDK4-R | GAAGGCACTGGCTTTTTGAG |
| ANGPTL4-F | TAGAGTCCCTGAAGGCCAGA |
| ANGPTL4-R | AATGAGCTGGGTCATCTTGG |

**Supplementary table 2 Top 15 up- and down-regulated DEGs in the GSE114007 dataset**

| **Name** | **Description** | **log2FoldChange** | **pval** | **up/down** |
| --- | --- | --- | --- | --- |
| POSTN | periostin | 6.4871827 | 7.05E-20 | up |
| COL1A1 | collagen type I alpha 1 chain | 5.7791765 | 1.76E-21 | up |
| HBB | hemoglobin subunit beta | 5.6144444 | 6.09E-13 | up |
| PENK | proenkephalin | 4.7030431 | 7.23E-11 | up |
| LRRC15 | leucine rich repeat containing 15 | 4.5668243 | 1.84E-17 | up |
| AMTN | amelotin | 4.440991 | 1.88E-09 | up |
| ASPM | assembly factor for spindle microtubules | 4.3602249 | 2.94E-18 | up |
| TGFBI | transforming growth factor beta induced | 4.1615329 | 1.64E-17 | up |
| GRIA2 | glutamate ionotropic receptor AMPA type subunit 2 | 4.1168459 | 2.55E-20 | up |
| ST6GALNAC5 | ST6 N-acetylgalactosaminide alpha-2,6-sialyltransferase 5 | 4.1162067 | 2.12E-21 | up |
| TNFSF15 | TNF superfamily member 15 | 4.0926563 | 6.57E-12 | up |
| TOP2A | DNA topoisomerase II alpha | 3.9919763 | 3.00E-13 | up |
| MKI67 | marker of proliferation Ki-67 | 3.8751723 | 1.24E-12 | up |
| TNFAIP6 | TNF alpha induced protein 6 | 3.8702343 | 9.74E-13 | up |
| THY1 | Thy-1 cell surface antigen | 3.8235759 | 4.49E-15 | up |
| CDKN1A | cyclin dependent kinase inhibitor 1A | -2.9924257 | 9.48E-22 | down |
| JUN | Jun proto-oncogene, AP-1 transcription factor subunit | -3.0350998 | 6.38E-26 | down |
| SLC38A3 | solute carrier family 38 member 3 | -3.0620676 | 1.51E-23 | down |
| SIK1 | salt inducible kinase 1 | -3.0848934 | 2.35E-14 | down |
| CSRNP1 | cysteine and serine rich nuclear protein 1 | -3.1490829 | 5.55E-20 | down |
| MAFF | MAF bZIP transcription factor F | -3.1604609 | 3.42E-58 | down |
| CISH | cytokine inducible SH2 containing protein | -3.295637 | 2.94E-44 | down |
| ATF3 | activating transcription factor 3 | -3.476798 | 1.00E-23 | down |
| ELF3 | E74 like ETS transcription factor 3 | -3.4826509 | 1.01E-17 | down |
| DDIT4 | DNA damage inducible transcript 4 | -3.4833897 | 5.71E-20 | down |
| FOSB | FosB proto-oncogene, AP-1 transcription factor subunit | -3.5043406 | 5.58E-12 | down |
| KIT | KIT proto-oncogene, receptor tyrosine kinase | -3.5308087 | 4.20E-16 | down |
| HILPDA | hypoxia inducible lipid droplet associated | -3.814199 | 2.30E-36 | down |
| ADM | adrenomedullin | -4.2694023 | 5.90E-33 | down |
| RND1 | Rho family GTPase 1 | -5.1228906 | 2.44E-16 | down |

**Supplementary table 3 Top 15 up- and down-regulated DEGs in the GSE169077 dataset**

| **Name** | **Description** | **log2FoldChange** | **pval** | **up/down** |
| --- | --- | --- | --- | --- |
| COL1A1 | collagen type I alpha 1 chain | 3.8541692 | 1.95E-06 | up |
| HLA-DRA | major histocompatibility complex, class II, DR alpha | 3.8295033 | 1.20E-05 | up |
| MT1X | metallothionein 1X | 3.674943 | 1.15E-04 | up |
| MMP13 | matrix metallopeptidase 13 | 3.5779243 | 6.13E-06 | up |
| COL1A2 | collagen type I alpha 2 chain | 3.5178871 | 5.26E-06 | up |
| CXCL8 | C-X-C motif chemokine ligand 8 | 3.3273241 | 3.83E-03 | up |
| COL9A1 | collagen type IX alpha 1 chain | 3.2331858 | 1.08E-04 | up |
| BMP1 | bone morphogenetic protein 1 | 3.2058096 | 1.92E-05 | up |
| EIF3F | eukaryotic translation initiation factor 3 subunit F | 3.1646661 | 1.13E-04 | up |
| POSTN | periostin | 3.1147353 | 1.70E-05 | up |
| SERPINF1 | serpin family F member 1 | 2.9914425 | 1.69E-05 | up |
| ALOX5 | arachidonate 5-lipoxygenase | 2.9778315 | 2.43E-04 | up |
| EPHB2 | EPH receptor B2 | 2.9773612 | 1.99E-05 | up |
| MXRA5 | matrix remodeling associated 5 | 2.9371539 | 3.82E-05 | up |
| SSX2IP | SSX family member 2 interacting protein | 2.8706694 | 1.32E-04 | up |
| TXNIP | thioredoxin interacting protein | -2.9796177 | 3.19E-05 | down |
| SLC7A8 | solute carrier family 7 member 8 | -3.0913136 | 8.16E-05 | down |
| GPM6B | glycoprotein M6B | -3.1038614 | 7.84E-05 | down |
| FKBP5 | FK506 binding protein 5 | -3.1051326 | 2.06E-04 | down |
| ADM | adrenomedullin | -3.135009 | 1.77E-05 | down |
| MAOA | monoamine oxidase A | -3.1375336 | 2.65E-04 | down |
| PDE2A | phosphodiesterase 2A | -3.1926711 | 3.72E-05 | down |
| SAA2 | serum amyloid A2 | -3.2358759 | 7.24E-05 | down |
| PDK4 | pyruvate dehydrogenase kinase 4 | -3.2808595 | 4.36E-05 | down |
| PCK1 | phosphoenolpyruvate carboxykinase 1 | -3.5108794 | 2.16E-05 | down |
| CYP4B1 | cytochrome P450 family 4 subfamily B member 1 | -3.5717458 | 6.52E-05 | down |
| HILPDA | hypoxia inducible lipid droplet associated | -3.578469 | 2.15E-06 | down |
| TMOD1 | tropomodulin 1 | -3.6322803 | 1.78E-05 | down |
| TSC22D3 | TSC22 domain family member 3 | -3.6651674 | 1.03E-06 | down |
| DDIT4 | DNA damage inducible transcript 4 | -4.2480746 | 7.82E-08 | down |
