# Supplementary material for: PDK4 inhibits osteoarthritis progression by activating the PPAR pathway
Source: J Orthop Surg Res. 2024 Feb 2;19:109. doi: 10.1186/s13018-024-04583-5 (PMC10835968; doi:10.1186/s13018-024-04583-5)

Supplementary figure 1 The read counts in the GSE114007 and GSE169077 datasets were normalized for each sample. A. GSE114007. B. GSE169077.


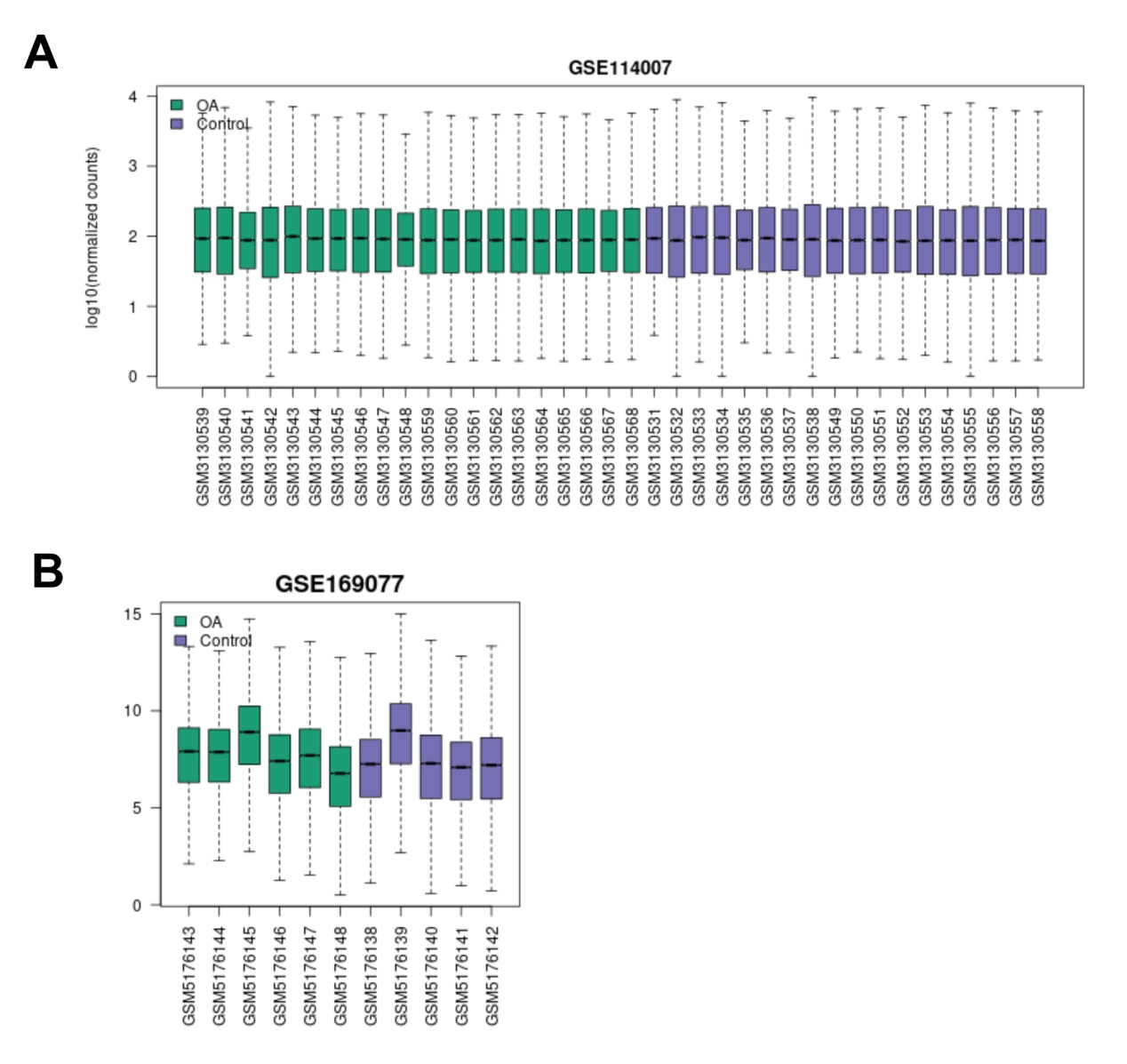


Supplementary Figure 2 Enrichment analysis of common DEGs. A. The top 5 items of the GO enrichment analysis, including BP, CC, and MF were depicted in the form of a bubble plot. B. DEGs highly enriched in the top 8 KEGG pathways were illustrated as a bar graph.
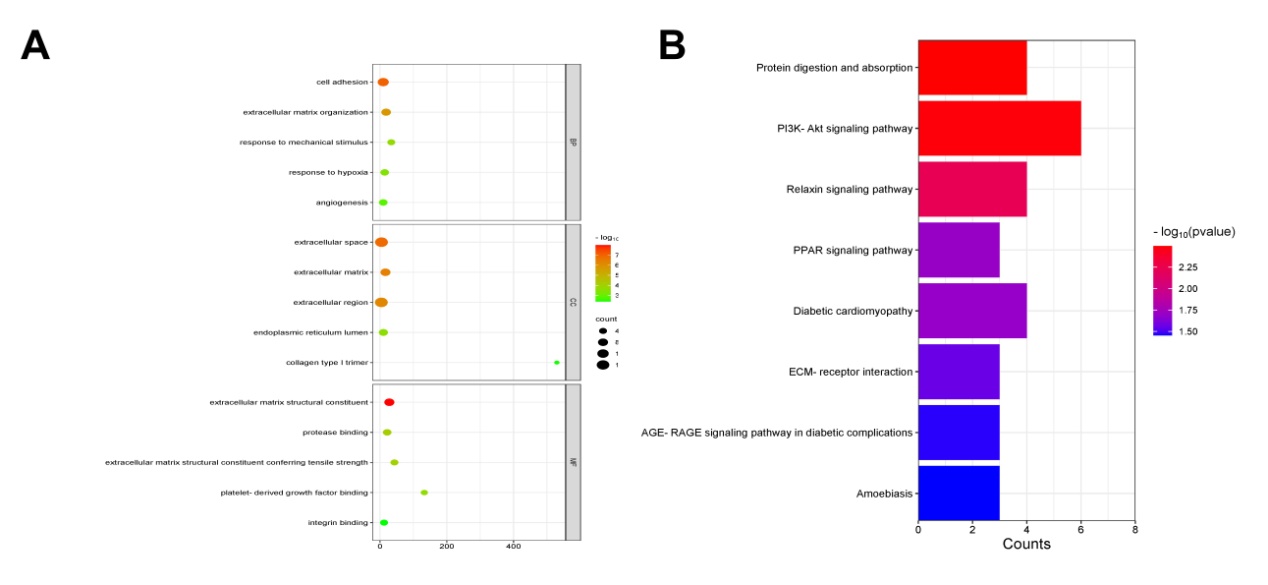


Supplementary Figure 3 A PPI network was established based on 39 DEGs using the STRING tool.
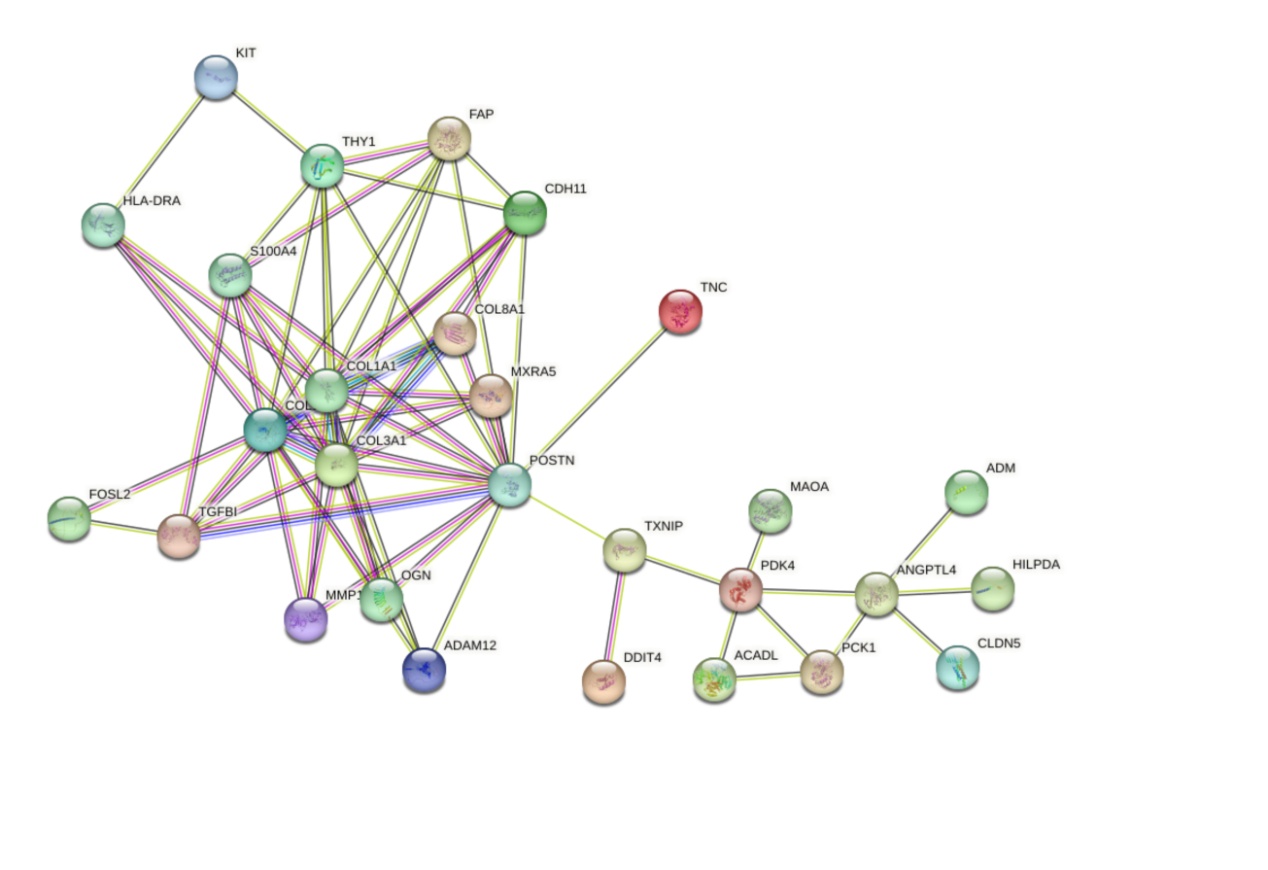


Supplementary Figure 4 Expression distribution and GO analysis of hub genes. A. Ridge line plot of the expression of hub genes. The horizontal coordinate represents the gene expression, the shape of the peaks indicates the dispersion between a set of data, and their height is the number of samples corresponding to the gene expression. B. Circle plot of GO enrichment analysis of hub genes.
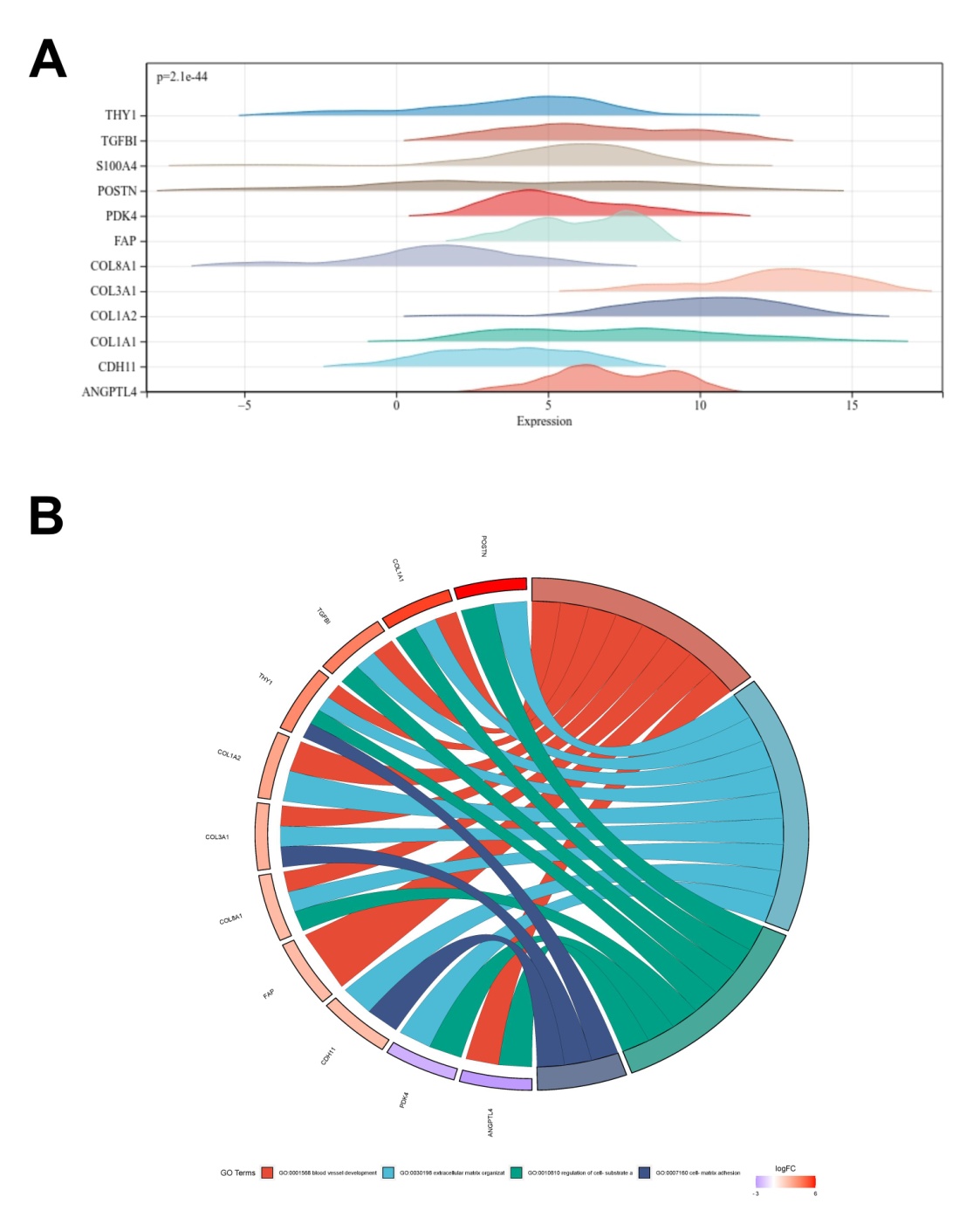


Supplementary Figure 5 ROC curves of hub genes were analyzed in the GSE114007 dataset. A. ANGPTL4. B. CDH11. C. COL1A1. C. COL1A1. D. COL3A1. E. COL8A1. F. FAP. G. PDK4. H. POSTN. I. S100A4. J. TGFBI. L. THY1.
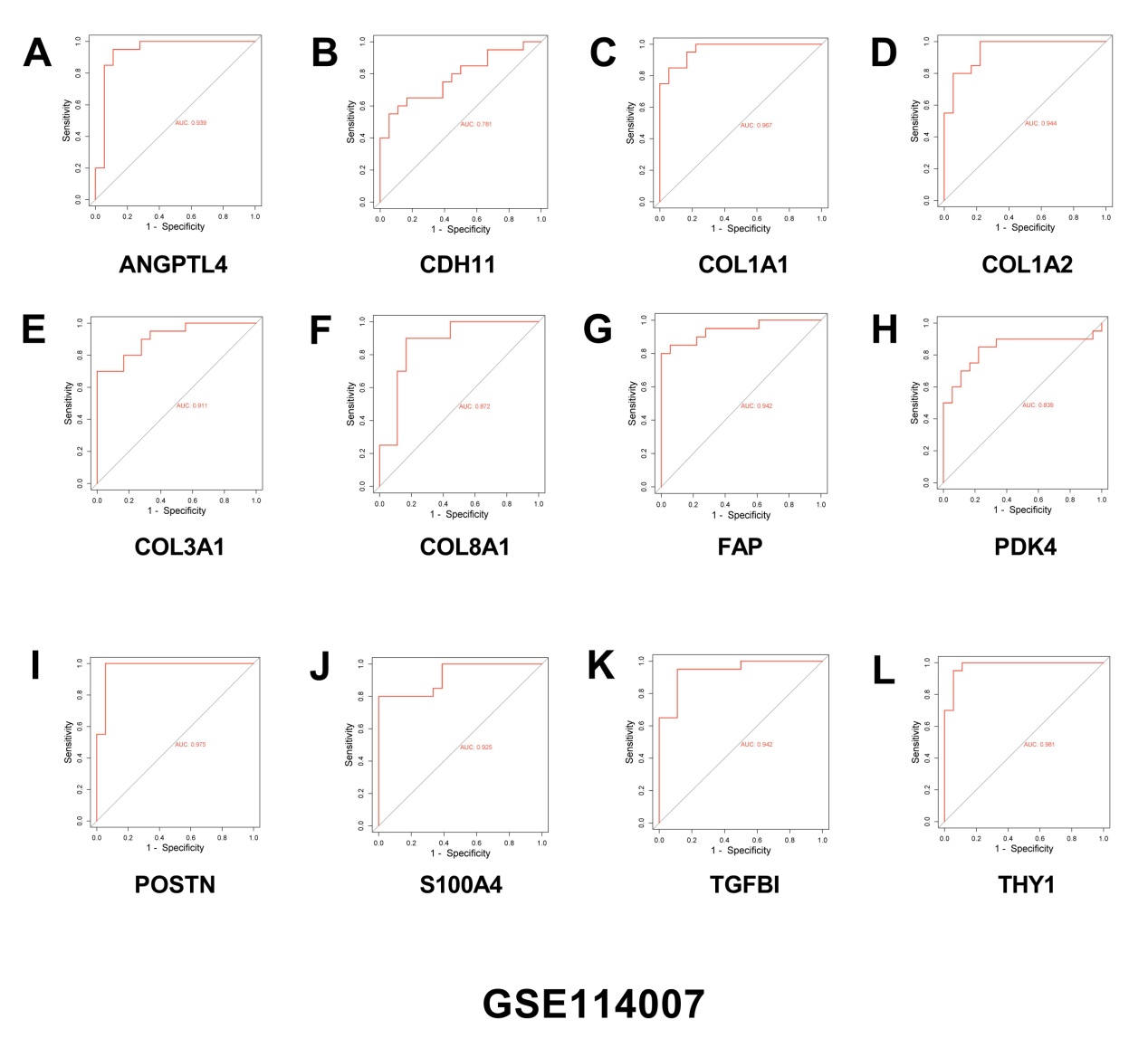


Supplementary Figure 6 ROC curves of hub genes were analyzed in the GSE169077 dataset. A. ANGPTL4. B. CDH11. C. COL1A1. C. COL1A1. D. COL3A1. E. COL8A1. F. FAP. G. PDK4. H. POSTN. I. S100A4. J. TGFBI. L. THY1.


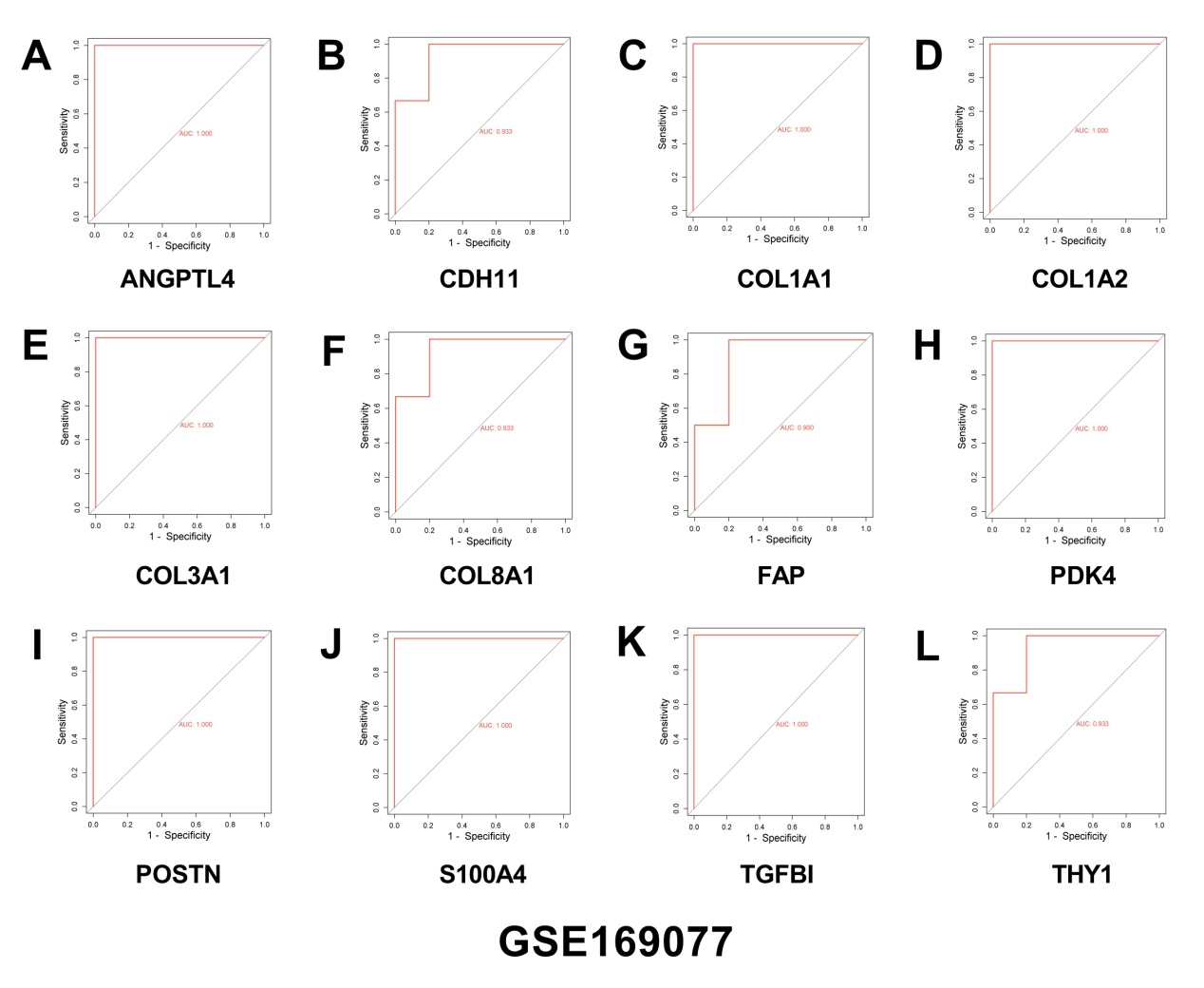

Supplement: Supplementary file 2 — Additional file 2. Supplementary figures for bioinformatics analysis of DEGs. [file 13018_2024_4583_MOESM2_ESM.docx]
